# Persistent Dizziness in an Older Patient Due to Vitamin B12 Deficiency-Related Subacute Combined Degeneration (SCD) After Correction of Hyponatremia: A Case Report

**DOI:** 10.7759/cureus.97605

**Published:** 2025-11-23

**Authors:** Yoshinobu Tabata, Junya Ohara, Yoshinori Ryu, Ryuichi Ohta

**Affiliations:** 1 Community Care, Unnan City Hospital, Unnan, JPN

**Keywords:** 80 and over, aged, cyanocobalamin therapeutic use, dizziness, general medicine, proprioception disorders, rural, subacute combined degeneration, vitamin b 12 deficiency

## Abstract

An 82-year-old woman presented with persistent dizziness that began one month earlier. Initial testing revealed severe hyponatremia (Na 104 mEq/L), and she was hospitalized for evaluation and fluid restriction, which normalized sodium levels. However, her dizziness persisted. The neurological examination revealed deep sensory impairment and lower motor neuron findings, without upper motor neuron signs. Laboratory testing revealed a markedly reduced vitamin B12 level (<148 pg/mL). Although spinal MRI showed no characteristic lesions of subacute combined degeneration (SCD), vitamin B12 deficiency was suspected as the underlying cause of her unsteadiness, and intravenous cyanocobalamin (1000 µg/day for 21 days) was administered. Her vibration sense improved, and she regained partial stability. This case highlights that vitamin B12 deficiency can manifest primarily as deep sensory disturbance without typical upper motor neuron signs or radiological findings, particularly in older adults. Excessive focus on concurrent metabolic abnormalities, such as hyponatremia, may delay recognition of neurological causes of dizziness. Clinicians should consider vitamin B12 deficiency or early SCD in elderly patients presenting with unexplained unsteadiness and perform thorough neurological assessments to enable timely treatment and prevent irreversible nerve damage.

## Introduction

Subacute combined degeneration (SCD) of the spinal cord is a neurological disorder, primarily caused by vitamin B12 deficiency [1]. It involves demyelination and axonal loss, predominantly affecting the white matter of the posterior and lateral columns of the spinal cord, leading to subacute sensory-motor ataxia and spastic paraplegia. Vitamin B12 deficiency becomes more common with age, affecting approximately one in 10 individuals aged 75 years or older [2,3]. Although SCD is rare, its symptoms typically begin with sensory disturbances in the hands, followed by reduced vibration sense, ataxic gait, and pathological reflexes such as hyperreflexia and a positive Babinski sign [4]. The diagnosis is supported by characteristic findings, including macrocytic anemia, low serum vitamin B12 levels, and abnormal results on gastroscopy or spinal MRI [5]. Treatment consists of intramuscular injections of cyanocobalamin, hydroxocobalamin, or mecobalamin at a dose of 1,000 µg daily for one week, followed by weekly injections for one month, and then monthly maintenance therapy [3].

However, older patients, commonly those older than 65 years, often present with vague or atypical symptoms in conditions associated with vitamin B12 deficiency [6]. Here, we report the case of an 82-year-old woman who presented with dizziness as the chief complaint. Despite the absence of upper motor neuron signs, pathological reflexes, or typical spinal MRI findings, she was clinically diagnosed with SCD and treated promptly. This case highlights the diagnostic challenges and therapeutic considerations of SCD in older patients.

## Case presentation

An 82-year-old woman presented to a rural hospital with progressive unsteadiness, appetite loss, and memory loss. One month before admission, she had developed gait instability that gradually worsened. Two weeks before admission, she complained to her family of persistent dizziness and a “foggy head,” with appetite loss, for which she consulted her primary care physician. She was prescribed betahistine mesylate (18 mg) daily, but her symptoms did not improve. Concerned about the persistence of her unsteadiness, her family brought her to the Department of General Medicine at a rural community hospital.

Initial laboratory evaluation revealed severe hyponatremia with a serum sodium concentration of 104 mEq/L (reference range: 135-150 mEq/L). Hyponatremia was suspected to be the cause of her dizziness, and she was admitted for further investigation and management. Her past medical history included hypertension, dyslipidemia, and right adrenal enlargement, with no prior neurological disorders. Her husband had passed away three months earlier. Her regular medications included febuxostat (10 mg), hydrochlorothiazide (12.5 mg), atenolol (25 mg), aspirin (100 mg), vonoprazan (10 mg), and rosuvastatin calcium (2.5 mg) daily.

On admission, her vital signs were stable: Glasgow Coma Scale 15, body temperature 36.4°C, heart rate 70 beats per minute, blood pressure 139/76 mmHg, respiratory rate 17 breaths per minute, and oxygen saturation 99% on room air. Physical examination revealed no signs of dehydration, peripheral edema, or volume overload.

Laboratory findings showed a serum osmolarity of 220.3 mOsm/L, urine osmolarity of 251 mOsm/L, urinary sodium of 40 mEq/L, and a fractional excretion of uric acid (FEUA) of 14.3% (Table 1).

**Table 1:** Initial laboratory data of the patient FEUA: fractional excretion of uric acid. Laboratory data indicate hypo-osmolar hyponatremia with inappropriately concentrated urine and elevated urinary sodium, suggesting the coexistence of syndrome of inappropriate antidiuretic hormone secretion (SIADH) and thiazide-induced hyponatremia.

Parameter	Patient's level	Reference
White blood cells (× 10^3^/μL)	6.7	3.5–9.1
Neutrophils (%)	81.2	44.0–72.0
Lymphocytes (%)	9.2	18.0–59.0
Hemoglobin (g/dL)	12.9	11.3–15.2
Hematocrit (%)	35.9	33.4–44.9
Mean corpuscular volume (fl)	108.8	79.0–100.0
Platelets (× 10^4^/μL)	15.8	13.0–36.9
Total protein (g/dL)	7.7	6.5–8.3
Albumin (g/dL)	4.1	3.8–5.3
Total bilirubin (mg/dL)	1.1	0.2–1.2
Aspartate aminotransferase (IU/L)	33	8–38
Alanine aminotransferase (IU/L)	20	4–43
Lactate dehydrogenase (U/L)	234	121–245
Blood urea nitrogen (mg/dL)	14.5	8–20
Creatinine (mg/dL)	0.65	0.40–1.10
Serum Na (mEq/L)	104	135–150
Serum K (mEq/L)	3.1	3.5–5.3
Serum Cl (mEq/L)	69	98–110
Uric acid (mg/dL)	2.1	2.0-7.0
Blood osmotic pressure (mOsm/L)	220.3	275-290
Urine test	-	-
Leukocyte	Negative	Negative
Protein	Negative	Negative
Blood	Negative	Negative
Urine osmolality (mOsm/L)	251	500-800
Urine sodium (mmol/L)	40	40-220
FEUA (%)	14.3	5-12

The laboratory data demonstrated hypokalemia, which was considered to be caused by appetite loss. Based on these findings, syndrome of inappropriate antidiuretic hormone secretion (SIADH) and thiazide-induced hyponatremia were considered likely etiologies. Neurological examination revealed a positive Romberg’s sign, absent Achilles tendon reflexes bilaterally, absent Babinski signs, and impaired vibration sense (medial malleolus: right 2 s/left 1 s; lateral malleolus: right 3 s/left 1 s), suggesting deep sensory deficits consistent with a lower motor neuron lesion.

**Figure 1:**
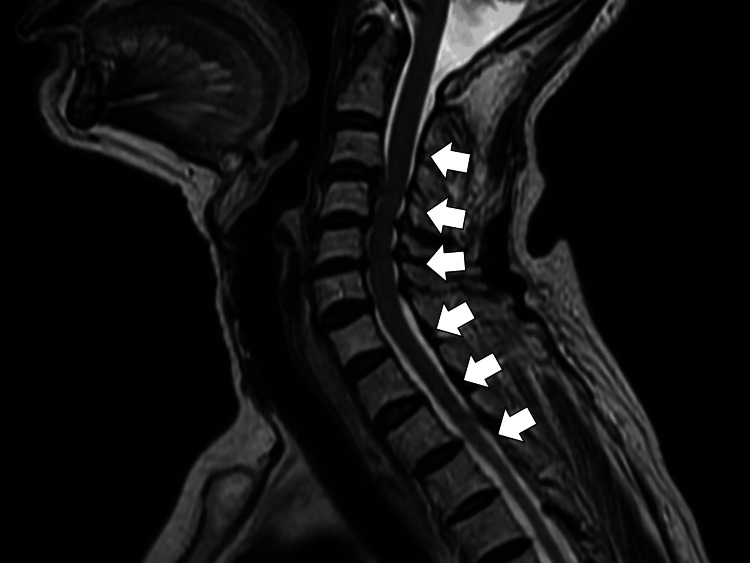
Cervical spinal magnetic resonance imaging (MRI) T2-weighted images of the cervical spine did not demonstrate any such abnormal signal intensity in the posterior columns, at the C3-C5 levels.

Hydrochlorothiazide was discontinued, and fluid restriction was initiated. By day seven of hospitalization, her serum sodium and potassium levels had normalized to 133 mEq/L and 4.1 mEq/L, respectively; however, her unsteadiness persisted. On day 14, serum vitamin B12 testing revealed a markedly low level (<148 pg/mL). Cervical spine magnetic resonance imaging (MRI) was performed to evaluate for SCD but showed no abnormal T2-weighted hyperintensities in the posterior columns (Figure 1).

No upper motor neuron signs were detected, making a definitive diagnosis of SCD difficult.

**Figure 2:**
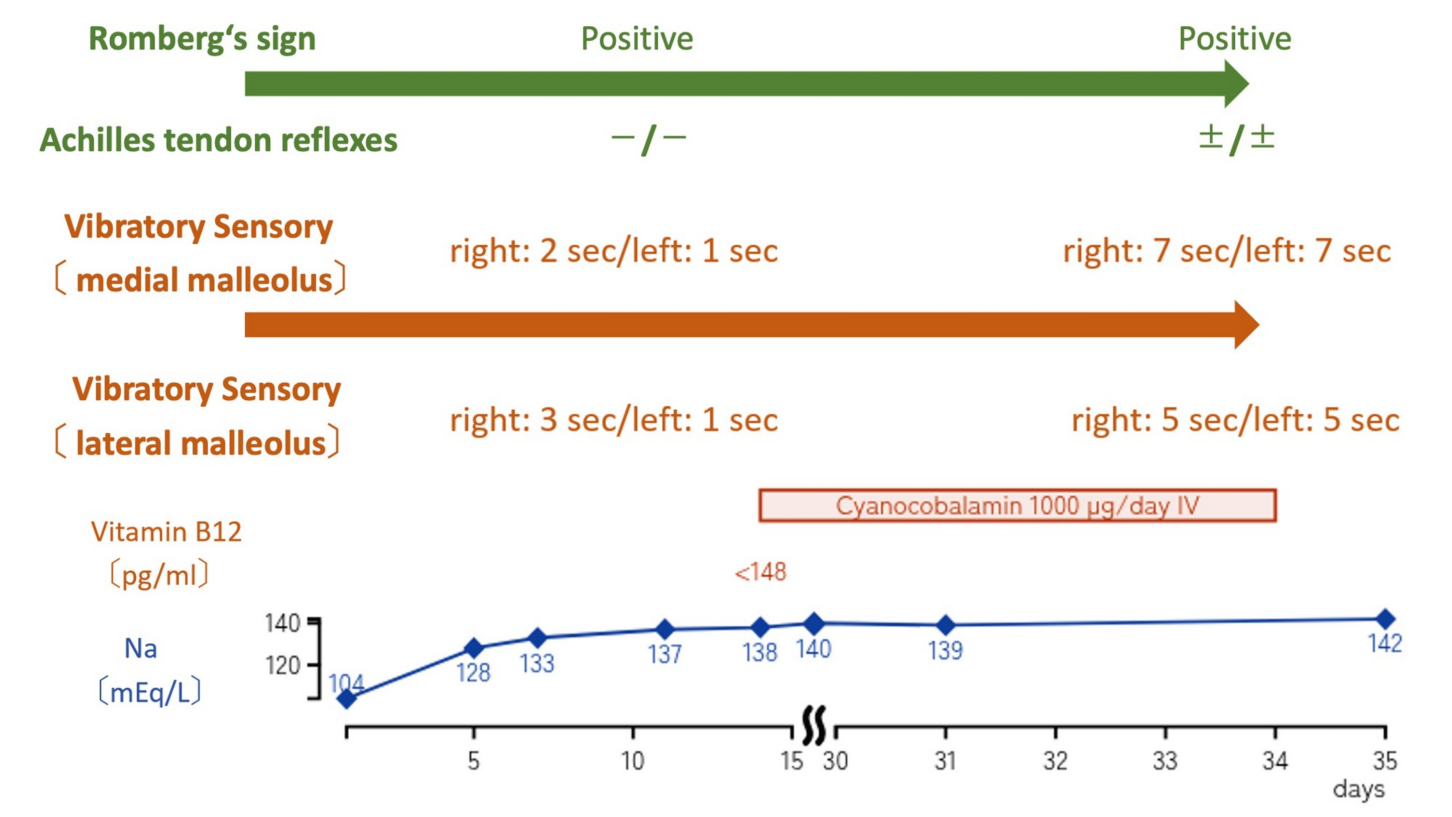
Clinical course of neurological findings and laboratory parameters during hospitalization The patient’s serum sodium (Na) level normalized within one week after admission, while serum vitamin B12 remained markedly low (<148 pg/mL). Intravenous cyanocobalamin (1,000 µg/day) was administered for 21 days (orange box). Despite the normalization of sodium levels, neurological symptoms persisted initially. Over the course of vitamin B12 supplementation, vibratory sensation at the medial and lateral malleoli gradually improved, and partial recovery of Achilles tendon reflexes was observed. Romberg’s sign remained positive throughout the observation period but showed functional improvement with rehabilitation.

Given her low vitamin B12 level and neurological findings, a deep sensory disturbance secondary to vitamin B12 deficiency was considered the cause of her symptoms. Intravenous cyanocobalamin (1,000 µg/day) was administered for 21 days. Following treatment, neurological re-evaluation demonstrated improvement in vibration sense (medial malleolus: right 7 s/left 7 s; lateral malleolus: right 5 s/left 5 s) and partial recovery of Achilles tendon reflexes (±/±), although the Romberg’s sign remained positive and she was unable to stand with her feet together. The patient was subsequently transferred to the hospital’s rehabilitation unit and discharged home after 30 days of hospitalization (Figure 2).

## Discussion

This case involved vitamin B12 deficiency accompanied by deep sensory disturbance, but did not meet all classical diagnostic criteria for SCD. However, as both SCD and vitamin B12 deficiency share the same therapeutic approach, a clinical diagnosis of suspected SCD secondary to vitamin B12 deficiency was made. The patient’s initial presentation of hyponatremia diverted attention from her primary complaint of unsteadiness, resulting in delayed evaluation of the underlying neurological cause. Because dizziness and unsteadiness in older adults have multiple potential etiologies, a comprehensive neurological assessment is crucial to prevent diagnostic oversight.

In older patients, the diagnosis of SCD or vitamin B12 deficiency should be primarily clinical, rather than dependent solely on laboratory or radiological findings. In this case, despite markedly low vitamin B12 levels and deep sensory impairment, there were no findings of anemia, upper motor neuron signs (such as hyperreflexia or a positive Babinski sign), or abnormal signal intensities in the posterior columns of the cervical spinal cord on MRI [7]. Previous studies have reported that approximately 25% of patients with vitamin B12 deficiency-related neuropathy do not exhibit macrocytic anemia [8]. Therefore, the absence of hematological abnormalities should not exclude the diagnosis of SCD.

Additionally, spinal MRI has limited sensitivity in detecting early or mild SCD. Reports indicate that a significant proportion of cases show no detectable spinal cord lesions, and excessive reliance on imaging may delay treatment [9,10]. Linazi et al. reported that abnormal MRI findings were observed in only eight of 42 SCD cases, underscoring the low sensitivity of MRI for this condition [11]. While radiological and upper motor neuron findings can support the diagnosis, overdependence on these features may postpone necessary intervention. The absence of upper motor neuron involvement in the present case may reflect either early-stage disease or the heterogeneous progression of vitamin B12-related neuropathy.

Neuropathy due to vitamin B12 deficiency primarily affects the posterior columns of the spinal cord, and as it advances, can involve large-diameter myelinated fibers (Aβ) in the peripheral nerves [12]. These fibers transmit proprioceptive, vibratory, and discriminative sensory input; their dysfunction results in impaired vibration sense and diminished deep tendon reflexes. As SCD typically develops subacutely, the patient’s one-month history of unsteadiness before admission is consistent with the expected disease course [13]. The absence of upper motor neuron signs in this case may also be related to the limited detectability of subtle deficits in older adults. Although nerve conduction studies or somatosensory evoked potentials can identify abnormalities when the spinal MRI appears normal, their clinical utility in elderly patients is limited, and prompt empirical treatment with vitamin B12 is generally preferable to invasive diagnostic testing.

Non-specific symptoms in older adults are often related to vitamin deficiencies, including vitamin B12 deficiency, which can present atypically as dizziness or gait instability [14-16]. Dizziness is a frequent complaint in the elderly, yet its underlying cause remains unidentified in approximately 12% of cases [17]. This diagnostic uncertainty arises from the wide range of potential etiologies, such as muscle weakness, balance disorders, and nutritional deficits. As demonstrated in this case, systematic differential diagnosis and timely initiation of therapy can improve patient outcomes and quality of life, particularly in the context of multidisciplinary general medicine care in rural hospitals [18-20]. Therefore, when older patients present with unsteadiness or dizziness, careful neurological examination and early consideration of vitamin B12 deficiency or SCD are essential to ensure appropriate and timely management.

Furthermore, this case highlights the importance of considering targeted screening and preventive nutritional strategies for older adults, particularly those over 60-70 years of age who frequently present with non-specific neurological symptoms. Age-related physiological changes, polypharmacy, reduced gastric acidity, and chronic atrophic gastritis increase the risk of vitamin B12 malabsorption in this population [6-8]. Several epidemiological studies have demonstrated that subclinical vitamin B12 deficiency is prevalent among community-dwelling older adults and remains undetected until neurological symptoms emerge [6-8]. Routine assessment of B-complex vitamin adequacy, either through periodic serum measurements or dietary evaluation, may help identify individuals at risk before irreversible neurological impairment develops. In settings such as rural hospitals, where older adults often present with dizziness, gait disturbances, or unexplained sensory deficits, implementing simple nutritional screening practices can contribute to the earlier detection and timely treatment of vitamin B12 deficiency.

## Conclusions

In this case, an elderly patient presented with persistent dizziness that initially appeared to result from hyponatremia but was ultimately caused by vitamin B12 deficiency. Despite the absence of typical radiological or upper motor neuron findings, neurological examination revealed deep sensory impairment consistent with early SCD. Prompt recognition and treatment with cyanocobalamin improved her symptoms. This case highlights that vitamin B12 deficiency should be considered in older adults with unexplained dizziness or gait disturbances, even in the absence of classical signs. It highlights the importance of comprehensive neurological assessment beyond metabolic abnormalities.
